# Looking after your partner: sentinel behaviour in a socially monogamous bird

**DOI:** 10.7717/peerj.83

**Published:** 2013-06-04

**Authors:** Mark C. Mainwaring, Simon C. Griffith

**Affiliations:** 1 Department of Biological Sciences, Macquarie University, Sydney, NSW, Australia; 2 School of Biological, Earth & Environmental Sciences, University of New South Wales, Sydney, NSW, Australia

**Keywords:** Incubation, Zebra finch, Predation, Bi-parental care, Natural selection, Sentinels, Social monogamy, Taeniopygia guttata

## Abstract

Natural selection favours those individuals with effective anti-predator defences. The presence of sentinels is known to be an effective form of defence amongst stable groups of individuals within cooperative and polygynous breeding systems. However, the presence of sentinels in the more prevalent socially monogamous breeding systems remains overlooked as an important benefit of such partnerships. Here, we describe a study in which we examined the presence and effectiveness of sentinels in a wild population of the socially monogamous zebra finch (*Taeniopygia guttata*). We found that when experimentally approached by a human observer during incubation, birds flushed from their nests at significantly greater distances when their reproductive partner was acting as a sentinel than when the partner was absent. The distance at which birds flushed was not influenced by the approach direction of the human observer, the gender of the incubating bird, the presence of conspecifics, the habitat type or the size of the breeding colony. Our results indicate that sentinels are an effective anti-predator defence amongst socially monogamous birds, and may represent a neglected benefit of the formation of stable social partnerships in birds. We suggest that whilst recent work has focused on the sexual conflicts that occur between males and females in socially monogamous pairs, we should not lose sight of the benefits that individuals may gain from their partner.

## Introduction

Avoiding predation is an almost ubiquitous challenge for most species and natural selection favours those individuals with effective anti-predator defences (Ruxton, Sherratt & Speed, 2004; Caro, 2005). Longevity is an important determinant of lifetime reproductive success (Pettorelli & Durant, 2007) and accordingly, a wide range of sophisticated anti-predator defences have evolved amongst prey species (Caro, 2005). The presence of sentinels, whereby one individual assumes a prominent position and scans for predators whilst other individuals engage in some other activity, such as foraging, has evolved as an effective form of defence. Sentinel behaviours have been well described in species with stable social groups of birds and mammals within cooperative and polygynous breeding systems (Beletsky, Higgins & Orians, 1986; Knight & Temple, 1988; Hollén, Bell & Radford, 2008; Ridley, Raihani & Bell, 2010; Clutton-Brock et al., 1999). Indeed, amongst cooperatively breeding birds and mammals, the deployment of sentinels as a common form of anti-predator defence has long been perceived as a classic example of kin selection or reciprocal altruism (Caro, 2005; Ridley, Nelson-Flower & Thompson, 2013). Even in circumstances in which sentinels are believed to be acting in their own individual interests (Clutton-Brock et al., 1999), other group members benefit from their presence. When sentinels are present, group members enjoy increased foraging success (Hollén, Bell & Radford, 2008) and receive early warnings of approaching predators (Caro, 2005).

Surprisingly, given the cooperation between a male and female that is inherent at the heart of a socially monogamous relationship, unequivocal evidence of sentinels in socially monogamous breeding systems remains elusive, despite some earlier reports of their presence (Morton & Shalter, 1977; Tilson, 1980; D’Agostino, Giovinazzo & Eaton, 1981). Over the past couple of decades, most research on socially monogamous species has focused on aspects of conflict rather than cooperation. For example, there has been much effort invested in questions relating to genetic infidelity (Petrie & Kempenaers, 1998; Griffith, Owens & Thuman, 2002), the conflict over parental investment (Parker, Royle & Hartley, 2002; Royle, Hartley & Parker, 2002), and maternal effects, particularly from the conflict-centric differential allocation hypothesis (Burley, 1988; Gil et al., 1999; Pryke & Griffith, 2009). Despite this apparent neglect, cooperative behaviour between partners such as sentinelling should be expected to have evolved within socially monogamous breeding systems (Caro, 2005) where the evolutionary fitness of one’s partner is linked to one’s own fitness. When one member of the pair has a low probability of detecting a predator but a high probability of receiving advanced warning of a predator from the other pair member, then active sentinelling behaviour should be advantageous. Effective sentinelling within partnerships may improve partner survival and also the efficiency with which they engage in activities such as foraging or incubation.

The purpose of this study was to experimentally test for the presence and effectiveness of sentinels in a socially monogamous bird, the wild zebra finch (*Taeniopygia guttata*). Although domesticated zebra finches are a well-used model system in studies of sexual conflict (Burley, 1988; Gil et al., 1999; Royle, Hartley & Parker, 2002), in the wild, zebra finches form life-long genetically monogamous pair bonds (Zann, 1996; Griffith et al., 2010) with high levels of behavioural synchrony (Mariette & Griffith, 2012; Mariette & Griffith, 2013). Individuals increase their fitness by breeding with a familiar partner (Adkins-Regan & Tomaszycki, 2007) and, as the long-term evolutionary interests of both parents overlap significantly, cooperative anti-predator behaviours should be expected (Ruxton, Sherratt & Speed, 2004; Caro, 2005). Whilst foraging, adult zebra finches suffer high mortality rates from aerial predators, such as Australian hobbies (*Falco longipennis*) and collared sparrowhawks (*Accipiter fasciatus*) (Zann, 1996), and are vulnerable in their enclosed bottle-shaped nests to predation by cats and a number of snake, lizard and bird species (Zann, 1996; Griffith, Pryke & Mariette, 2008). Consequently, in this study, we tested the hypothesis that, when approached by a human observer during incubation, birds would flush from their nests at a higher rate and significantly greater distances when their reproductive partners were present and acting as a sentinel than when they were absent.

## Methods

### Study site and general methods

Data were collected between September–December 2012 from wild zebra finches breeding in nine colonies within the Fowlers Gap Arid Zone Research Station located in far-western New South Wales, Australia (31°05’S, 42°42’E). The study site is characterised by open mixed chenopod and acacia shrubland (Griffith, Pryke & Mariette, 2008).

A total of 287 nestboxes were available for breeding zebra finches to occupy across the nine colonies that varied in size from 2 to 42 active nests within an area of approximately 100 square metres. From early September, all nestboxes were checked at least every other day for the commencement of breeding and egg laying. We were interested in examining anti-predator behaviour directed towards the partner specifically rather than towards offspring, and therefore targeted birds during the incubation phase, i.e., in the absence of hatched nestlings. In wild zebra finches, incubation usually commences when the last egg is laid (Zann, 1996; Gilby, Mainwaring & Griffith, in press) and, counting the day that the last egg was laid as day zero, we quantified sentinel behaviour on the fifth day of incubation. We choose a single day of the incubation cycle to standardise our data and reduce any variation that may have been caused by changes in investment over the reproductive cycle. We chose day five because in an earlier study we had previously recorded incubation duration times in detail for both males and females on this day (Gilby, Mainwaring & Griffith, in press), enabling us to control for natural departures (see below).

### Quantifying sentinel behaviour

We quantified the presence and effectiveness of sentinel behaviour of the birds in response to the standardised approach of a human observer towards the nest during trials at 205 nests. The direction in which the human observer approached a nestbox was systematically alternated between the ‘front’, thereby potentially allowing the incubating bird to see and hear the approaching human and the ‘side’ at an angle of 90° to the front of the box, thereby potentially allowing the incubating bird to hear, but not see, the approaching human. By conducting this work on individuals breeding inside nestboxes we were able to ascertain that the bird sitting in the nest was unable to see the experimenter. Birds in natural nests that are constructed out of grass stems may well have been able to see out of the side. On the day prior to trials, a Garmin Etrex GPS was used to mark the starting point, either 100 m in front or to the side of the focal nestbox, for the subsequent day’s trial. The starting points were marked as waypoints in the GPS allowing the observer to find the starting point on the day of the trial without disturbing the birds at the nestbox.

For each trial, once the observer was in position at the starting point, the trial started (at time zero) and the observer then set off towards the box at a random departure time between 0–90 s from the start time. This randomised start time allowed us to control for random natural departures by the birds from the nestbox and also separate the effect of being in the vicinity of a nest (standing at the start point) and being a direct threat (approaching). The average incubation bout of wild zebra finches on day five of incubation is 23 min (Gilby, Mainwaring & Griffith, in press), and therefore we would only expect a low number of incidents where the bird was naturally leaving the box during the total period of our trials (i.e., trials lasted a maximum of 3 min and 10 s and birds only leave the box once every 23 min normally). At the departure time, the observer walked slowly at a constant speed of one metre per second directly towards the focal nestbox whilst using binoculars (Leica: 8 × 42 BN) to aid visual identification of behaviour. The observer recorded the appearance of a bird in the doorway of a nestbox (as they flushed off the eggs and prepared to leave the box) and recorded the distance (metres from the box) and time (seconds taken to walk to the box) at which the bird flew from the nest box. The gender of the incubating bird was also recorded. Meanwhile, the presence and gender of the sentinel bird, which was usually conspicuously associated with the nestbox, was also recorded. Sentinel birds were typically perched in a tree or bush within about 3 m of the nestbox, and their presence or absence was ascertained both at the beginning and throughout the trials. The presence or absence of other conspecifics seen during the trial was recorded and the habitat type was classified as being either ‘open’ if acacia bushes were absent or thinly distributed or ‘closed’ if acacia bushes were abundant. The size of the breeding colony, defined as the number of pairs breeding actively (having eggs or nestlings in the nest) in the colony at the time of the trail, was also quantified. Of the 205 trials, there were 10 trials in which neither bird was observed, thereby leaving 195 trials where birds were observed. All of the trials were conducted by one person (MCM).

### Statistical analyses

The data were analysed in the SPSS v20.0 statistical package. We began by using a General Linear Model (GLM) to examine the relationship between the distance from the nest and the time from the nest at which birds flushed from nestboxes during trials, where the dependent variable was ‘flight distance’ (0–100 m) and the fixed explanatory covariate term was ‘flight time’ (0–100 s).

We examined variation in ‘flight distance’ using a GLM. The dependent variable was ‘flight distance’ (0–100 m), the fixed explanatory factorial terms were ‘approach direction’ (front or side), ‘focal bird sex’ (male or female), ‘partner present’ (yes or no), ‘conspecific present’ (yes or no) and ‘habitat type’ (open or closed), and the fixed explanatory covariate term was ‘colony size’ (number of pairs in the colony). The maximal model included all of the explanatory variables, and their two-way interaction terms, and the explanatory variables were all assessed for significance when they were the last terms in the model. Non-significant effects were sequentially removed by stepwise deletion until the most parsimonious model was obtained (Crawley, 1993). We used the stepwise deletion procedure of model selection as previous studies have shown it to perform equally as well as other methods of selecting predictive models (Murtaugh, 2009).

We then used a GLM to examine the relationship between the total time from the beginning of each trial (which consisted of the sum of the random departure time between 0–90 s and the time from when the observer started walking to when the incubating bird flushed between 0–100 s) and the random departure time, where the dependent variable was ‘total time’ (0–190 s) and the fixed explanatory covariate term was ‘random departure time’ (0–90 s). The means are presented ± 1 standard error and a critical P-value of 0.05 is applied throughout.

## Results

**Figure 1:**
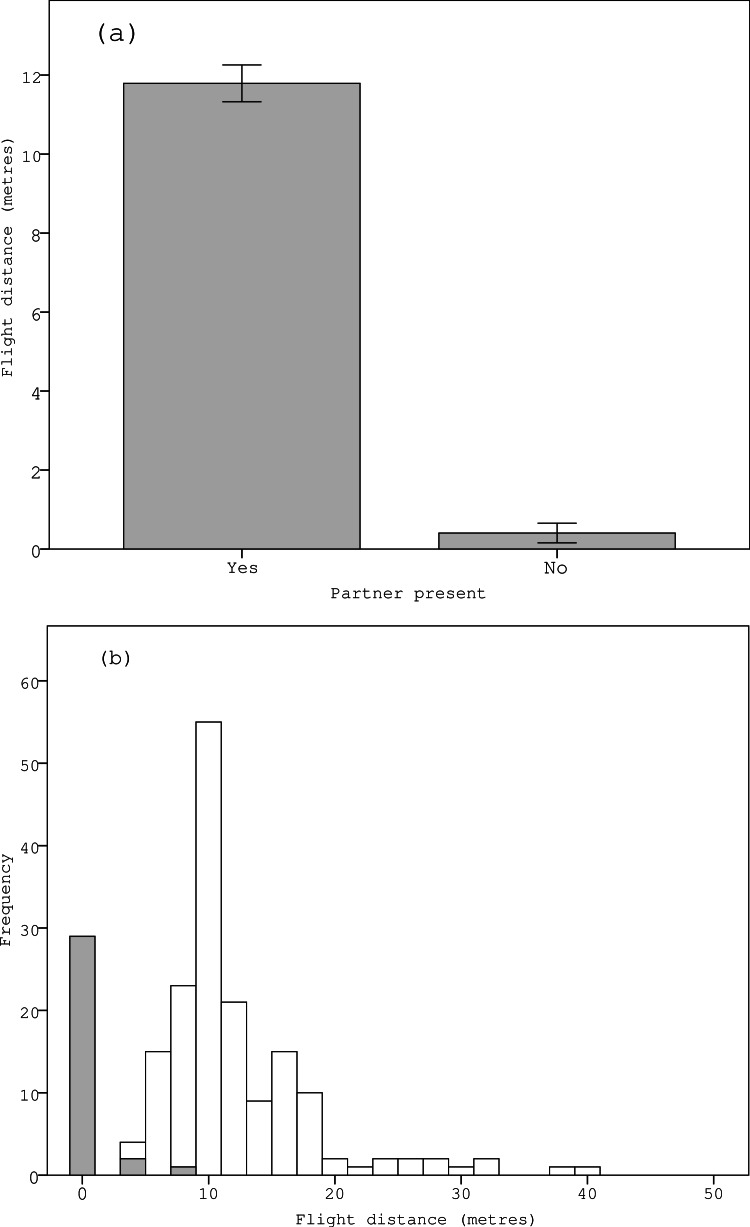
Variation in the (A) mean and (B) relative frequency of ‘flight distances’ at which incubating zebra finches flushed when approached by a human observer in relation to the presence of their reproductive partner acting as a sentinel. Note that in figure (B), the grey bars represent those trials in which the partner was absent and the white bars represent those trials in which the partner was present.

Sentinel birds were observed in 163 (84%) of the 195 trials in which an individual was inside the nest incubating the eggs, and not observed during 32 (16%) of the trials. In the 163 trials in which sentinels were observed, males were found to be incubating in 67 (41%) trials (i.e., the sentinel was a female in these cases) and in the 32 trials without sentinels, males were found to be incubating in 17 (53%) trials. There was a strong positive correlation between the observer’s distance from the nest and the time taken by the observer to walk to the nest for those trials when the incubating bird left the nest (*F*_1,194_ = 2698.255, *P* < 0.001), which meant that the observer was indeed moving towards the nest at a steady pace. In the 163 trials in which sentinel birds were present, incubating birds flushed from the nest when the observer was 11.8 ± 0.5 m from the nest, whereas in the 32 trials in which sentinel birds were absent, the observer was essentially able to catch the bird in the nest as incubating birds flushed from nest when the observer was only 0.4 ± 0.2 m from the nest. This means that incubating birds flushed from their nests when the observer was a significantly greater distance from the nest when their reproductive partner was present and observed acting as a sentinel (*P* < 0.001) (Table 1) (Figs. 1A and 1B). Further analyses indicated that there was a strong positive correlation between the total time from the beginning of each trial (which consisted of the sum of the random departure time between 0–90 s and the time from when the observer started walking to when the incubating bird flushed between 0–100 s) and the random departure time (*F*_1,194_ = 2297.197, *P* < 0.001). This indicates that departure times by incubating birds were driven by the observer’s proximity to the nest rather than being random natural departures. Meanwhile, the distance at which incubating birds flushed from nests was not influenced by the approach direction of the observer, the gender of the focal bird, the presence or absence of conspecifics, the type of habitat, the size of the breeding colony or any of their two-way interaction terms (Table 1).

**Table 1:** Summary of a General Linear Model examining variation in the ‘flight distance’ of incubating zebra finches when approached by a human observer. Note that non-significant interactions are not included for reasons of brevity.

Explanatory term	Effect size	*F*-value (*df* = 1, 195)	*P* value
Partner present	0.487	95.043	<0.001
Colony size	0.010	2.011	0.158
Habitat type	0.010	2.001	0.159
Approach direction	0.005	0.959	0.329
Focal bird sex	0.00003	0.063	0.803
Conspecific present	0.00003	0.063	0.803

## Discussion

We found that acting as a sentinel for an incubating partner was a common and effective form of anti-predator defence in the socially monogamous zebra finch. When a human observer approached nests during incubation, birds flushed from their nests at significantly greater distances when their partner was acting as a sentinel than when their partner was absent. We are confident that incubating birds did not flush as a result of their directly seeing the approaching experimenter because there was no difference in the flushing distance between those trials in which the observer approached from the front or the side. In the latter situation, a bird on the nest could not see through the side wall of the nestbox, whereas an observer approaching from the front could be seen through the nest box entrance hole.Therefore, by focusing on birds that were visually occluded by the nest box itself we have been able to demonstrate that individuals benefit from the presence of a sentinelling partner. Nevertheless, we believe that sentinel behaviour will be just as effective in birds in natural nests and certainly we see no reason to believe that it will have increased in frequency in this nest box nesting population. Although the nestboxes were attached to steel picket posts they were always positioned within a few metres of an acacia or wattle bush (Griffith, Pryke & Mariette, 2008) and sentinel birds were seen perching on the nearest bush, and within a few metres of the nest, providing them with good opportunity to scan for predators.

The sentinelling behaviour that we have observed will have enabled the adult in the nest to leave the nest before potentially being surprised and trapped by a predator. We have previously observed one direct predation of an adult bird being consumed after being caught on a natural nest by an eastern brown snake (*Pseudonajat extilis*), and found half a dozen more active natural nests that appear to have been pulled apart by predators and in which we have seen large numbers of adult feathers, providing suggestive evidence that an adult was predated from the nest (Griffith, Pryke & Mariette, 2008). An adult taken by surprise in a nest is very vulnerable. The nest is a light construction of thin grass stems readily broken apart by most predators, and it is hard for adults to escape as the only exit is the funneled entrance hole, scarcely wider than the bird itself. Exiting the nest before an approaching predator reaches the nest is therefore the best survival strategy. Our results demonstrate the effectiveness of this strategy and indeed on the 32 occasions when there was no sentinel, the observer reached the nestbox and was able to trap and capture the bird inside the nest.

Zebra finches do not mob, attack, and attempt to distract predators and instead, either flee or freeze to keep out of harm’s way (Zann, 1996). Even if the eggs or nestlings are vulnerable to a predator, the adults will not make any attempt to dispel the predator. Therefore, we can be reasonably confident that the sentinel behavior we observed is focused on preserving the life of the partner rather than being an incidental response to an anti-predator call that might have another functional response such as eliciting help from conspecifics to mount a mobbing response. For that reason, although we only studied the behavior on a single day of the incubation period, we expect such sentinelling to be maintained across the whole reproductive period, and indeed to occur throughout non-breeding periods as well. We believe that the primary function of the behavior is to maintain the partnership rather than the reproductive attempt itself. In this, our work contrasts nicely with one of the observations of sentinel behaviour in a non-cooperatively breeding bird – the red-winged blackbird. In this polygynous species, male sentinels gain direct fitness benefits by warning offspring about an approaching predator and/or instigating a mobbing defence against a predator (Beletsky, Higgins & Orians, 1986; Knight & Temple, 1988; Yasukawa, Whittenberger & Nielsen, 1992; Burton & Yasukawa, 2001). This sentinelling behaviour in the red-winged blackbird reduces predation on eggs or nestlings and is actively selected by females as one of their mate choice criteria because good males will provide better defence for their offspring (Knight & Temple, 1988). Zebra finches use sentinelling to protect a partner, whereas red-winged blackbirds appear to primarily use sentinel behaviour to protect offspring. However, when comparing our findings with those from the blackbirds, it is important to remember that the data of the two studies were collected during the incubation and nestling phases respectively. Sentinel behaviour (that is focused on the survival of the brood) might be expected to change throughout the reproductive cycle as investment in the brood increases and this will be worthy of future study.

Sophisticated forms of anti-predator defences such as sentinelling are expected to evolve in social species that suffer high levels of predation and live in habitats that facilitate some individuals with a good vantage point to sense approaching danger (Bednekoff, 2001; Caro, 2005). These criteria apply in the zebra finch, which typically live in small social groups and forage on the ground in semi-open habitat where they are vulnerable to aerial ambush predators such as Australian hobbies or collared sparrowhawks. Whilst sentinelling for each other will probably make sense throughout the whole year it makes particular sense during reproduction when one adult is required to spend time alone in the nest. Previous work has demonstrated that pairs have very synchronised behaviour and move around together almost habitually and visit the nest together during nestling provisioning (Mariette & Griffith, 2012). We recently found that the pairs that had the highest levels of behaviour synchrony (spending more time in each other’s company) hatched a higher proportion of their eggs resulting in higher fitness (Mariette & Griffith, 2012; Mariette & Griffith, 2013). Perhaps this may, in part, be due to a greater efficacy of incubation by individuals that are reassured by the presence of their partner on guard outside the nest. Another important determinant of fitness that has been demonstrated in a study of domesticated zebra finches was that pairs that have experience of breeding together are more efficient at initiating a reproductive attempt once conditions allow (Adkins-Regan & Tomaszycki, 2007). These two lines of evidence provide good motivation for a partner to look out for its incubating mate.

The costs of acting as a sentinel are not necessarily great and sentinelling is no longer seen as an altruistic behaviour in the way that it once was (Clutton-Brock et al., 1999; Wright et al., 2001; Wright, Maklakov & Khazin, 2001; Bednekoff & Woolfenden, 2003; Ridley, Raihani & Bell, 2010; but see Ridley, Nelson-Flower & Thompson, 2013). The incubation phase is one of the few times when an individual will find itself potentially physically separated from its mate, and alone in the environment (Zann, 1996; Elie et al., 2010). Isolating an individual zebra finch from other conspecifics (visually and acoustically) has been shown to be physiologically stressful (Perez et al., 2012), and therefore perhaps sentinels are partly motivated by a desire to remain in contact with their partner. Furthermore, taking up a sentinelling position should reduce an individual’s predation risk because a sentinel can choose a position that is near cover and provides good sight lines to approaching danger (Clutton-Brock et al., 1999).

The mechanism through which the incubating bird learned of a potential predator is currently unclear, although there are three possibilities. First, the sentinelling bird may have given an alarm call alerting the incubating bird. Second, the cessation of an ongoing duet between the pair (Elie et al., 2010) may have alerted the incubating bird to the possibility of danger, causing it to have approached the nestbox entrance to check for itself. Third, the incubating bird may have heard the flapping wings of the fleeing partner as this has previously been shown to be an effective anti-predator strategy in other species (Hingee & Magrath, 2009). Moreover, given that sentinel and other anti-predator behaviours have been shown to be plastic over spatial and temporal timescales, the cues observed in the zebra finches may differ between different contexts (Caro, 2005; Ridley, Nelson-Flower & Thompson, 2013).

Over the past few decades, much research effort in socially monogamous animals has focused on aspects of sexual conflict, and the zebra finch is one of the most widely used models in this area (Burley, 1988; Gil et al., 1999; Royle, Hartley & Parker, 2002; Forstmeier et al., 2011). We believe that our findings help to redress the balance a little and highlight the mutual benefits that a male and female provide for one another in the social bond of monogamy. Recent work on the wild zebra finch has demonstrated that pairs are reproductively faithful to one another (Griffith et al., 2010), are behaviourally coordinated to a high degree (Mariette & Griffith, 2012; Mariette & Griffith, 2013), and have subtle and highly ritualised acoustic communication (Elie et al., 2010). This emerging picture of the social bond of the wild zebra finch does contrast with much of the work focused on the domesticated zebra finch revealing various aspects of sexual conflict. However, we believe that the unpredictable ecological conditions of the Australian arid zone (Morton et al., 2012), and the selective and behavioural pressure exerted by predators helps to reinforce the strength and depth of the social bond in wild zebra finches.

Social monogamy is the predominant mating bond formed in birds (>90% of species, (Lack, 1968)), and the behavioural and physiological dynamics at the heart of this pair bond play an important role in life-history evolution. Recently breeding pairs were described as a ‘flock of two’ (Lima, 2009), and our findings support the idea that the individuals in a socially monogamous partnership have the opportunity to benefit mutually in a number of ways that effect their own individual fitness. Although sentinels are most classically observed in cooperatively breeding mammals and birds (Clutton-Brock et al., 1999; Ridley, Raihani & Bell, 2010) as one of the clearest manifestations of highly social and cooperative behaviour, our work demonstrates that it may be found in a much broader context.

We believe that our findings should lead to a renewed interest in the cooperative aspects of social monogamy that have been neglected for so long in favour of the more fashionable ideas focused on the conflict between the partners. These conflicts over investment, and individual fitness, are important but have been overstated in recent decades. The strength and duration of the partnership between a male and female has the potential to directly affect individual fitness in a number of ways that have yet to be thoroughly explored and we believe that our findings will help to establish the importance of those determinants of individual fitness.

## Supplemental Information

10.7717/peerj.83/supp-1 Supplemental Information Raw data

## Additional Information and Declarations

### Field Study Permissions

The following information was supplied relating to ethical approvals (i.e. approving body and any reference numbers): The study was carried out with the knowledge and approval of the Director of the UNSW Fowlers Gap Arid Zone Research Station.
